# Changes in vital signs of hospitalized patients may not identify the need to escalate care as well as other bedside observations

**DOI:** 10.1016/j.resplu.2026.101529

**Published:** 2026-10-10

**Authors:** Christian Sebat, Frank Sebat, John Kellett, Daleen Penoyer

**Affiliations:** a Division of Pulmonary, Critical Care & Sleep Medicine, Department of Medicine, 4150 V Street, Suite 3400, University of California, Davis, Sacramento, CA 95817, United States; b Kaweah Delta Health, 400 W. Mineral King Ave, Visalia, CA 93291, United States; c Kritikus Foundation, Redding, CA, United States; d School of Clinical and Biomedical Sciences, University of Greater Manchester, Deane Road, Bolton BL3 5AB, United Kingdom; e Strategic Innovations, Orlando Health, 1126 S. Division Ave, Orlando, FL 328056, United States

**Keywords:** Failure to rescue, Early warning scores, Patient assessment, Rapid response systems, Vital signs, Bedside assessment, Signs and symptoms, Bedside examination

## Abstract

**Background:**

Most Rapid Response Team activations rely on vital signs, frequently aggregated into weighted scoring systems, such as the National Early Warning Score (NEWS). Nevertheless, a substantial proportion of avoidable in-hospital deaths still result from delayed recognition of evolving pathophysiology and under-appreciation of patient or family concerns.

**Aim:**

To compare the performance of NEWS with observations that can be rapidly assessed at the bedside without equipment or calculation, using death within 24 h as a surrogate marker for patients requiring immediate attention.

**Methods:**

Retrospective analysis of data collected prospectively on Rapid Response Team arrival to 4581 adult patients in general medical, surgical, or intermediate care units at a 450-bed regional medical center in California, USA.

**Results:**

Eight observations were identified that required no equipment to assess, which had a significant association with 24-h mortality; ranked by adjusted odds ratio these were (1) jaundice, (2) need for supplemental oxygen, (3) capillary refill time, (4) part or all of the body feeling cold, (5) objectively distressed breathing, (6) altered mentation, (7) dry skin, and (8) cyanosis. A logistic regression model of these 8 variables had a significantly higher discrimination for death within 24 h of Rapid Response Team arrival than NEWS (i.e., AUC of 0.790 SE 0.014 versus 0.707 SE 0.020, *p* < 0.0001).

**Conclusion:**

In patients who trigger a Rapid Response Team call, bedside information that can be obtained rapidly without equipment or calculation, may provide earlier and more reliable signals of illness severity than NEWS and other early warning scores based on traditional vital signs.

## Highlights

• Simple observations may help identify hospitalized patients needing immediate attention.
• The identification of these observations does not require calculations or equipment.
• In critically ill hospitalized patients these observations may outperform NEWS.

## Introduction

Many hospitals use early warning scores (EWS) and rapid response teams (RRTs) to ensure timely intervention for patients at risk of critical illness. The most widely validated system is the National Early Warning Score (NEWS), first introduced in 2012.^1^ NEWS combines vital signs, oxygen saturation, oxygen need, and mental status, requires no laboratory results, and was designed to predict 24-h mortality as a surrogate marker for patients who needed immediate attention.^2^

Despite the widespread adoption of EWS, avoidable deaths persist, often due to delayed recognition of other bedside observations—such as changes in appearance and behavior—that concern clinicians and families. Following the death of a 13-year-old girl from sepsis after concerns of her family were not acted on, Martha’s Rule has been introduced in the United Kingdom, which authorizes any alarmed individual—including family members—to initiate an urgent clinical review.^3^ Incorporating such intuitive concerns and other bedside observations into existing early warning systems may improve the early recognition of clinical deterioration, as demonstrated by the Dutch Early Nurse Worry Indicator Score (DENWIS),^4^ which has been shown to improve identification of patients at-risk for critical illness.^5^, ^6^, ^7^

To further explore the value of additional bedside observations as triggers of clinical concern and the process of gestalt, which allows clinicians to pull together multiple pieces of information to recognize or form an overall picture of what is going on with a patient,^8^, ^9^ we performed a retrospective secondary analysis of clinical data collected between 2013 and 2018. This database was obtained from a prior prospective observational study on the value of capillary refill time; it included the collection of a wide variety of observations about the patients’ appearance upon RRT arrival, which could be rapidly assessed at the bedside without the need for equipment and can be easily taught.^10^ The aim of this study was to compare the ability of the National Early Warning Score to predict 24-h mortality with these bedside observations in patients to whom a RRT has been called.

## Methods

### Setting

This study is a secondary analysis of data collected between March 2013 and March 2018 as part of a previously reported prospective observational study. The cohort included consecutive adult patients for whom a rapid response team was activated while hospitalized on general medical, surgical, or intermediate care units at a 450-bed regional medical center in California, USA.^10^ During the study period, 73,436 patients were admitted, and 6480 rapid response team activations occurred, corresponding to an activation rate of 88.2 per 1000 admissions.

### Study process

The standard of care at the study hospital required a comprehensive bedside assessment using a novel, bedside-focused surveillance and response system, which did not incorporate NEWS. For non-palliative patients, activation of the rapid response team was mandatory when two or more of eight weighted signs of vitality (Supplemental Fig. 1) were present.^11^ Observations recorded by the rapid response team nurse at the initial bedside assessment were entered into a predefined data collection form and stored in the database used for this analysis.

This anonymized study database contained patient age, sex, admission date, date and time of RRT call and team arrival, and for most patients, the date and exact time of death or hospital discharge. It also contained a complete set of vital signs, mental status, level of oxygen support and oxygen saturation performed upon arrival by the RRT before any intervention took place, from which NEWS was retrospectively calculated. In addition, the study data collection form specifically asked for “Breathing labored YES/NO?”, Accessory muscle use YES/NO?”, “Skin temperature – WARM or COLD?”, “Skin diaphoretic YES/NO”, and “Skin dry YES/NO?” As all these required a response, there was no missing data. If patients had either labored breathing or displayed use of accessory muscles, they were considered to have objective signs of distressed breathing. Pain levels were recorded on a scale from 1 to 10, altered or abnormal mental status was defined as Glasgow coma scale (GCS) <15 or not “alert-normal” by a 7-point level of consciousness scale (i.e., Agitation, Anxiety, Apathy, Alert-Normal, only responsive to Voice, Pain, or Unresponsive).^12^ CRT was measured by application of moderate pressure for 5 s or greater on the ventral tip of the index finger; after release of pressure, the nurse slowly counted (i.e., 1/1000, 2/1000, 3/1000, etc.) up to seven noting the number of seconds that elapsed before the tip of the finger returns to its previous color.^13^

The data collection form also prompted entry of free text descriptions of the patient’s visual appearance of the skin at the time of RRT arrival, which were captured in a “word cloud” (Supplemental Fig. 2). From this word-cloud we selected the observations of “cyanosis”, “ashen”, “blue”, “mottled”, “cyanosis/cyanotic” and “jaundice” for further analysis. We defined cyanosis as any description that included the terms “ashen”, “blue”, “mottled”, or “cyanosis/cyanotic”.

### Inclusion and exclusion criteria

All records of patients not receiving palliative care, which had sufficient plausible data recorded to calculate NEWS at the time of the RTT arrival and before any RRT directed intervention was provided, and the exact date and time of death after RTT arrival were included in the study. These criteria excluded 1899 patients from analysis (Fig. 1); these patients were younger (65.7 SD 17.8 versus 67.2 SD 17.3 years, *p* 0.001), more likely to die within 24-h (odds ratio 1.33, 95%CI 1.05–1.68), but no more likely to be transferred to ICU (odds ratio 1.03, 95%CI 0.92–1.15).

**Fig. 1:**
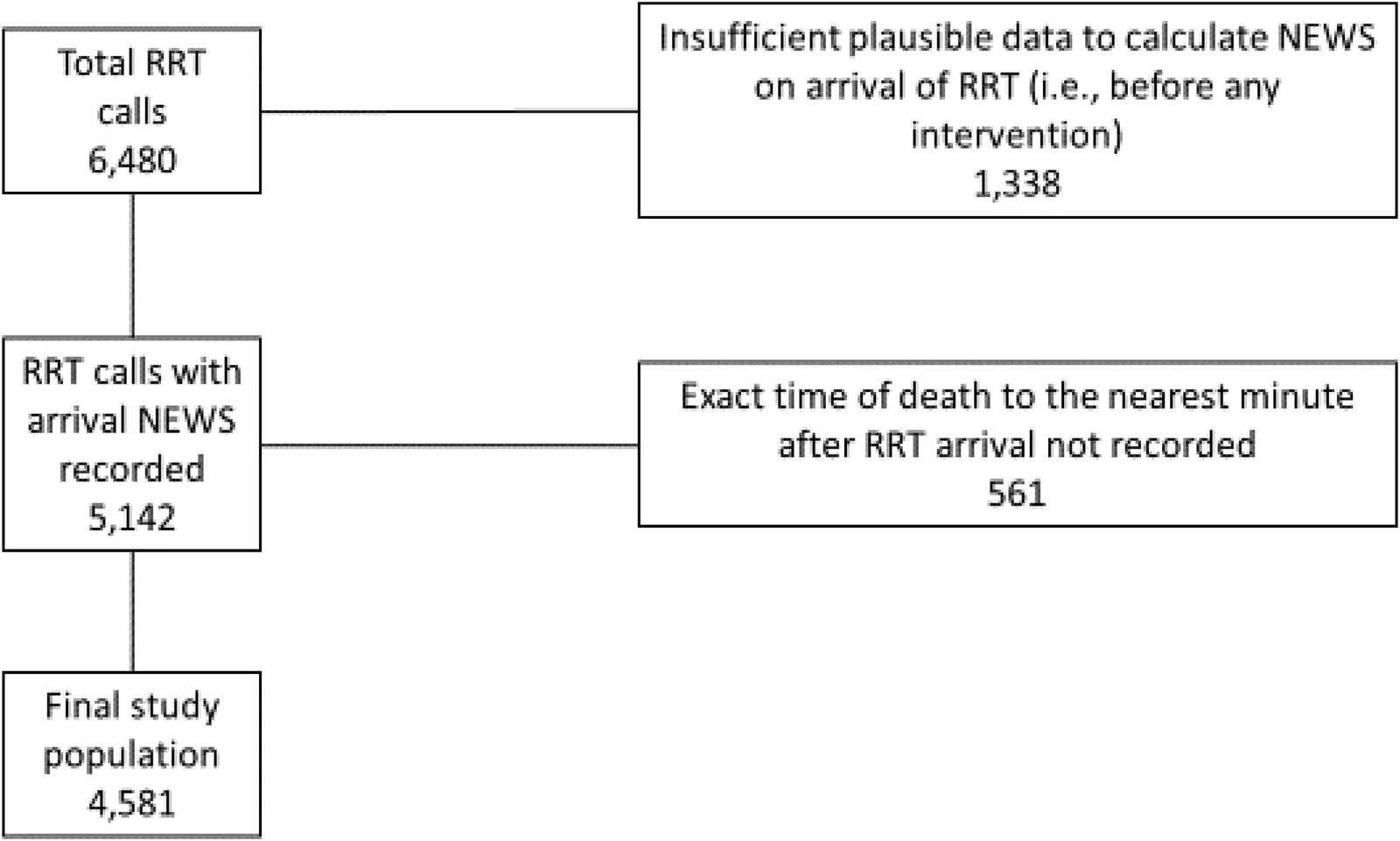
**Exclusion of patient data from the original study cohort of 6480 patients (see Ref.**^5^**) if there was not enough data to calculate the National Early Warning Score (NEWS), and if the exact time of the rapid response team (RRT) arrival and death were not recorded**.

### Missing data

There were no missing data in the patients included in the final study cohort. However, for the two observations extracted from the free-text descriptions (i.e., jaundice and cyanosis), we could not be certain these were absent if they were not recorded.

### Outcome

Death within 24-h of RRT arrival, used as a surrogate metric for patients who need immediate attention.

### Statistic methods and data analysis

The study complies with STROBE guidelines.^14^ All calculations were performed using Epi-Info version 6.0 (Center for Disease Control and Prevention, USA) and logistic regression analysis using Logistic software.^15^ Logistic regression was performed only on bedside observations in the dataset that were significantly associated with 24 h mortality and required no equipment to assess. The discrimination for mortality was determined by calculating the area under the receiver operating characteristic curve (AUC) using MedCalc statistical software (https://www.medcalc.org), which was compared by the method of Delong et al.^16^ Values of the AUC range from 0 to 1; 0.5 suggests no discrimination, 0.7–0.8 is considered acceptable, 0.8–0.9 is considered excellent, and more than 0.9 is considered outstanding.^17^ A normal distribution was assumed for all continuous variables,^18^ which were compared by using analysis of variance. We used Chi squared analysis with Yates’ continuity correction to show the association of categorical variables with 24-h mortality. The p-value for statistical significance was 0.05.

### Ethics

The study was conducted in accordance with the ethical standards of the responsible institutional review board and with the principles of the Declaration of Helsinki. The study protocol was reviewed and approved by the Kaweah Delta Healthcare District institutional review board. Study title: Value of Capillary Refill as Part of an Early Warning Score to Identify General ward Patients At Risk for Critical Illness. IRB # 328820, FWA # FWA00002487, approval date February 19th, 2019. Informed consent was waived.

## Results

Out of the final study population of 4581 patients attended by the RRT, 2269 (49.5%) were transferred to the intensive care unit, 1006 (22.0%) died in hospital of whom 224 (4.9% of all patients) died within 24 h of the RRT arrival. Patients who died within 24 h were significantly older (71.9 SD 15.6 versus 67.0 SD 17.4 years, *p* < 0.0001) and had been in hospital longer before the RRT was called (8.5 SD 13.4 versus 5.3 SD 9.2 days, *p* < 0.0001) than survivors (Table 1).

**Table 1:** Comparison of characteristics and interventions between patients who died within 24 h of RRT call and survivors.

**Patient characteristics and interventions**	**All patients**	**Alive 24-h after RRT call**	**Died within 24-h of RRT call (%)**	
***N***	***4581***	***4357***	***224***	***p***
Age (years)	67.2 SD 17.3	67.0 SD 17.4	71.9 SD 15.6	<0.0001
Length of hospital stay (days)	14.8 SD 18.8	15.1 SD 19.0	8.9 SD 13.4	<0.0001
Days to RRT call after admission	5.5 SD 9.5	5.3 SD 9.2	8.5 SD 13.4	<0.0001
Length of stay after RRT (days)	9.3 SD 14.7	9.8 SD 15.0	0.4 SD 0.3	<0.0001
Duration of RRT (minutes)	59.3 SD 94.0	59.2 SD 93.9	61.7 SD 94.8	NS
Heart rate (bpm)	104 SD 31	104 SD 31	103 SD 30	NS
Respiratory rate (bpm)	25.9 SD 9.2	25.7 SD 9.2	27.9 SD 9.6	<0.0001
Systolic blood pressure (mmHg)	12.8 SD 4.0	130 SD 39	108 SD 40	<0.0001
Temperature (°C)	37.0 SD 1.0	37.0 SD 1.0	36.7 SD 1.1	<0.0001
Oxygen saturation (%)	93.9 SD 6.9	94.0 SD 6.8	91.5 SD 9.2	<0.0001
FiO_2_	0.32 SD 0.14	0.32 SD 0.13	0.39 SD 0.19	<0.0001
NEWS	9.4 SD 3.2	9.3 SD 3.2	11.6 SD 2.7	<0.0001
Pain (1–10 scale)	1.9 SD 3.4	2.0 SD 3.4	1.5 SD 3.0	0.06
Number of interventions	1.70 SD 1.00	1.69 SD 1.02	1.95 SD 1.07	0.0002
Male sex	2328	2213	115 (4.9%)	NS
NEWS ≥5	4273	4050	223 (5.2%)	0.0002
No interventions	376	364	12 (3.2%)	NS
≥2 interventions	2440	2295	145 (5.9%)	0.0005
ICU transfer	2269	2115	154 (6.8%)	<0.0001
Oxygen given	3609	3428	181 (5.0%)	NS
Assisted ventilation (BiPAP)	168	153	15 (8.9%)	0.02
Volume expansion	1156	1078	78 (6.7%)	0.0009
Heart rate or rhythm control	440	426	14 (3.2%)	NS
Inotropes	191	161	30 (15.7%)	<0.0001
Blood pressure lowered	384	374	10 (2.6%)	0.04
IV diuresis	459	441	18 (3.9%)	NS
Sedation or pain control	771	733	38 (4.9%)	NS
Reversal of opiates or benzo-diazepam	209	189	20 (9.6%)	0.002

We identified ten bedside observations within our dataset, which could be easily taught, required, little time, and no equipment to assess. All of these, except pain and diaphoretic skin, were associated with 24-h mortality (Table 2). A logistic regression model of the remaining eight observations, confirmed all of them were significantly associated with 24-h mortality (Table 3), and had an AUC for 24-h mortality of 0.790 SE 0.014, significantly higher than that of NEWS, which had an AUC of 0.704 SE 0.018 (*p* < 0.0001) (Fig. 2). This eight-observation model had excellent goodness-of-fit (Hosmer-Lemeshow statistic 0.78, *p* 0.94).

**Table 2:** Odds ratio for 24-h mortality of observations that require no time or equipment to assess – ranked by odds-ratio.

**Observation**	**Odds ratio**	**95%CI**	**Chi-square**	***p***
Supplemental oxygen	4.88	(2.20–11.44)	19.67	<0.0001
Jaundiced	4.83	(3.15–7.39)	67.48	<0.0001
Capillary refill time >3 s	4.32	(3.25–5.74)	126.44	<0.0001
Altered mental status	3.96	(2.31–6.90)	31.09	<0.0001
Distressed breathing	3.93	(2.71–5.71)	64.74	<0.0001
All or part of body feels cold	3.51	(2.54–4.85)	69.25	<0.0001
Ashen, blue, mottled, or cyanotic	3.21	(2.29–4.51)	53.64	<0.0001
Skin feels dry	1.47	(1.00–2.17)	3.96	0.047
In pain	0.85	(0.63–1.14)	1.12	0.28
Diaphoretic skin	0.76	(0.50–1.15)	1.57	0.21

**Table 3:** Logistic regression of 8 bedside observations that were significantly associated with 24 h mortality and required no equipment to assess – ranked by coefficient and adjusted odds ratio.

**Observation**	**Coefficient (SE)**	Adjusted odds ratio (95%CI)*	***p***
Jaundice	1.582 (0.225)	4.87 (3.13–7.56)	<0.0001
Supplemental oxygen	1.161 (0.400)	3.19 (1.46–7.00)	0.0037
Capillary refill time >3 s	0.991 (0.155)	2.69 (1.99–3.65)	<0.0001
All or part of the body feels cold	0.929 (0.173)	2.53 (1.81–3.55)	<0.0001
Distressed breathing	0.907 (0.190)	2.48 (1.71–3.59)	<0.0001
Altered mental status	0.896 (0.270)	2.45 (1.44–4.16)	0.0009
Skin feels dry	0.662 (0.197)	1.94 (1.32–2.85)	0.0008
Ashen, blue, mottled or cyanotic	0.480 (0.187)	1.62 (1.12–2.33)	0.0100

* The odds ratios are adjusted odds ratios because they control for the other predictor variables included in the model.

**Fig. 2:**
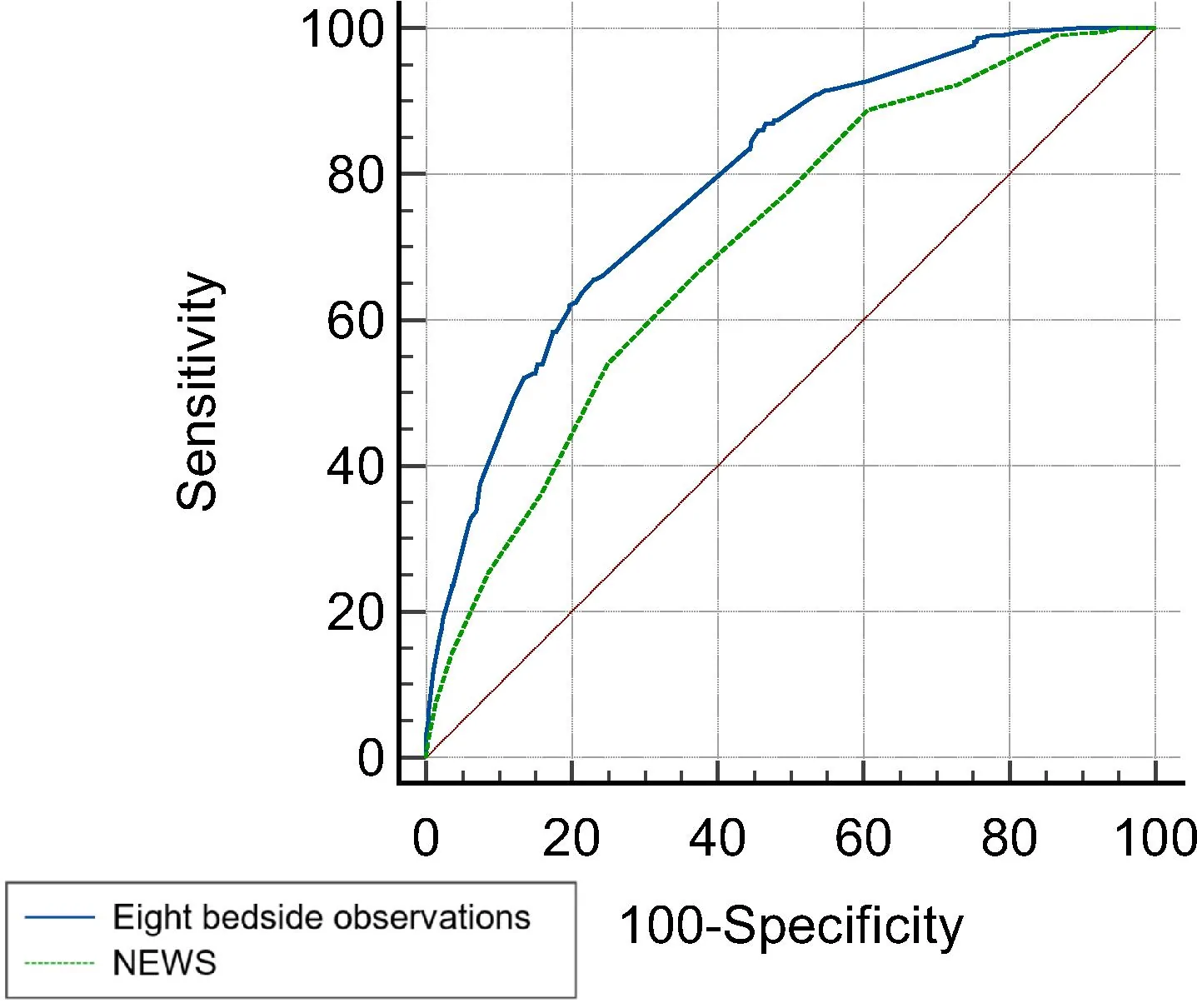
**The area under the receiver operating characteristic curve (AUC) of NEWS compared with the logistic regression model (**Table 3**) of eight bedside observations**.

Much simpler logistic regression models also had a better discrimination for 24-h mortality than NEWS. For example, a model with only four variables which included altered mentation, distressed breathing, CRT >3 s, and cold skin had AUC of 0.751 SE 0.016, which was significantly higher than NEWS (*p* 0.016); only 2 out of 520 (0.4%) patients who had none of these four variables died within 24 h, compared with 116 out of 857 (13.5%) with two or more.

## Discussion

### Main findings

This retrospective analysis of data from hospitalized patients to whom an RRT team was called found eight simple bedside observations (Table 3) that are easily taught, do not require calculations or equipment to measure and can identify patients who are likely to die within 24 h and therefore, in need of immediate attention. The study directly compared the prediction of imminent mortality by these eight observations with NEWS, which has to be calculated from measurements that take time and equipment operated by trained healthcare professionals to acquire^19^; the AUC of the eight observations was 0.790, compared with 0.707 for NEWS. Our findings are similar to those of a recent prospective study in emergency department patients, in which clinicians’ unstructured gestalt outperformed the Modified Early Warning Score (MEWS), in which the prediction of critical illness by clinicians’ gestalt had an AUC of 0.809, compared with 0.731 for MEWS.^20^

Despite CRT being influenced by ambient temperature and inter-observer variability,^21^ this study confirms its clinical value.^10^ Altered mental status, distressed breathing, cold skin and cyanosis are well established features of life-threatening illness and also included in the Dutch Early Nurse Worry Indicator Score DENWIS score.^5^ Not surprisingly jaundice had the highest association with mortality; jaundice from multiple causes has been reported in as many as 40% of ICU patients, and can indicate pre-existing liver disease, sepsis, multisystem organ failure, or a reflection of transient hypotension (shock liver), right-sided heart failure, the metabolic breakdown of red blood cells, or pharmacologic toxicity.^22^ We also found that dry, rather than diaphoretic skin, has a weak but significant association with imminent mortality, possibly because it may indicate severe dehydration.^23^

### Interpretation

In this study population the discrimination for 24-h mortality of the predictive model of 8 bedside observations was close to excellent (i.e., AUC 0.790 SE 0.014), whereas the discrimination of NEWS was barely acceptable (i.e., AUC 0.707 SE 0.017).^17^ It is well established that in most clinical circumstances NEWS identifies those patients who need to be seen urgently; out of over 8 million published worldwide observations only 0.06% of patients with a NEWS <5 die within 24 h, compared 1.78% of those with a NEWS ≥5.^24^ However, the patients in this study were a much sicker population, as its 24-h mortality was 4.9% and 93% had a NEWS ≥5.

### Clinical relevance

A number of bedside observations identified in this study demonstrated greater sensitivity for detecting impending clinical deterioration than traditional vital sign measurements. These observations are readily assessable, and education regarding their measurement and interpretation is not complex, supporting their potential utility in improving early identification of patients at risk for critical illness. Furthermore, these findings suggest that reliance on trends in conventional vital signs or their composite scores—even when augmented by continuous monitoring and machine learning approaches^25^—may be insufficient to reliably detect early deterioration. In contrast, alterations in mental status, objectively assessed respiratory effort, cutaneous findings, and capillary refill time may more directly reflect changes in tissue perfusion and oxygenation. Notably, emerging evidence indicates that skin mottling and capillary refill time are superior to lactate levels in assessing the response to sepsis therapy.^26^, ^27^

### Limitations

This study is a retrospective analysis of a study performed several years ago on very sick ward patients. Although some interventions and management protocols may have changed over time, it is unlikely that the clinical observations and vital sign derangements of critical illness will have altered much. The original study was performed in a hospital with a very high RRT call rate^28^ and a mature rapid response system, underpinned by years of staff training and organizational enculturation, which included training on the value of and how to measure CRT.^11^ It included a novel RRT calling mechanism with precisely defined criteria, requiring nurses to perform a comprehensive clinical assessment. As this unique setting may have led to overfitting of our predictive model,^29^ our findings may not apply to other patient populations in different clinical environments and need to be confirmed elsewhere.

More than 29% of patients to whom an RRT was called had missing values and were, therefore, excluded from this analysis. These patients may have included the sickest, who died before a full set of observations could be recorded. However, overall, patients with missing values were only slightly more likely to die, and no more likely to be admitted to ICU.

Our expert opinions unavoidably influenced the design of the data collection form, and the visual observation of the skin, i.e., jaundice and cyanosis, were derived from free text descriptions, and may have been present in more patients but not charted. Although some of the bedside patient observations identified by this study may be difficult to objectively quantify, and future studies may need to define terms such as altered mental status, dry, sweaty, cyanosed, cold etc., more precisely.^30^

## Conclusion

In patients who trigger an RRT call, bedside information that can be obtained rapidly without equipment or calculation, may provide earlier and more reliable signals of illness severity than NEWS and other EWS based on traditional vital signs. It is possible that other vital bedside information remains unidentified, and several variables identifiable by our senses should be more precisely defined and examined. The value of these observations needs to be tested in different patient populations and, if confirmed, more broadly adopted into patient assessments.

## Authors contributions

All authors contributed to the conception and design of the study (JK and FS), or acquisition of data (CS and FS), or analysis and interpretation of data (JK, FS, CS, DP). All authors were involved in drafting of the paper and approved the final version for submission. All authors agree to be accountable for all aspects of the work.

## Prior publication

There has been no prior publication in any form of this paper or the information it contains.

## Data availability statement

Anonymized data of this study are available to all interested parties upon request.

## CRediT authorship contribution statement

**Christian Sebat:** Writing – review & editing, Writing – original draft, Supervision, Methodology, Investigation, Conceptualization. **Frank Sebat:** Writing – review & editing, Writing – original draft, Visualization, Validation, Supervision, Project administration, Methodology, Investigation, Funding acquisition, Formal analysis, Data curation, Conceptualization. **John Kellett:** Writing – review & editing, Writing – original draft, Visualization, Validation, Supervision, Software, Methodology, Formal analysis, Data curation, Conceptualization. **Daleen Penoyer:** Writing – review & editing, Writing – original draft, Project administration, Methodology, Investigation, Conceptualization.

## Ethics declaration

This study was approved by the Kaweah Delta Healthcare District institutional review board.

IRB # 328820, FWA # FWA00002487. Informed consent was waved.

## Funding

This research did not receive any specific grant from funding agencies in the public, commercial, or not-for-profit sectors.

## Declaration of competing interest

None of the authors have any conflict of interest.

## Appendix A Supplementary material

Supplementary Data 1 Supplemental Figure 1: Kaweah Health Rapid Response Team Activation Criteria "10 signs of Vitality" Supplemental Figure 2: Word-cloud of the relative frequency of free-text descriptive terms of patient/subject appearance documented in data-collection sheet by the RRT nurse.

## References

[1] Holland M., Kellett J. (2023). The United Kingdom's national early warning score: should everyone use it? A narrative review. Intern Emerg Med.

[2] Prytherch D.R., Smith G.B., Schmidt P.E., Featherstone P.I. (2010). ViEWS–towards a national early warning score for detecting adult inpatient deterioration. Resuscitation.

[3] Welch J., Murkin J., Edwards E., Inada-Kim M., Subbe C. (2025). Giving patients, families and staff a reliable voice in acute care: expert guidance for implementation of Martha's rule in UK hospitals. Future Healthc J.

[4] Bonnamy J., Dart J., Palermo C., Molloy R., Small K., Connell C. (2026). 'They don't know my body': health consumer recognition and escalation of acute deterioration – a systematic review. BMJ Qual Saf.

[5] Douw G., Huisman-de Waal G., van Zanten A.R., van der Hoeven J.G., Schoonhoven L. (2016). Nurses' 'worry' as predictor of deteriorating surgical ward patients: a prospective cohort study of the Dutch-Early-Nurse-Worry-Indicator-Score. Int J Nurs Stud.

[6] Hwang J.I., Chin H.J. (2020). Relationships between the National Early Warning Score 2, clinical worry and patient outcome at discharge: retrospective observational study. J Clin Nurs.

[7] Douw G., Huisman-de Waal G., van Zanten A.R.H., van der Hoeven J.G., Schoonhoven L. (2017). Capturing early signs of deterioration: the Dutch-early-nurse-worry-indicator-score and its value in the Rapid Response System. J Clin Nurs.

[8] Cook C. (2009). Is clinical gestalt good enough?. J Man Manip Ther.

[9] Nemeth J., Kas P., Atkinson P. (2026). CJEM debate series: gestalt in emergency medicine: Judgment we should teach, or bias we should constrain?. CJEM.

[10] Sebat C., Vandegrift M.A., Oldroyd S., Kramer A., Sebat F. (2020). Capillary refill time as part of an early warning score for rapid response team activation is an independent predictor of outcomes. Resuscitation.

[11] Sebat F., Vandegrift M.A., Childers S., Lighthall G.K. (2018). A novel bedside-focused ward surveillance and response system. Jt Comm J Qual Patient Saf.

[12] Sessler C.N., Gosnell M.S., Grap M.J., Brophy G.M., O’Neal P.V., Keane K.A. (2002). The Richmond agitation-sedation scale: validity and reliability in adult intensive care unit patients. Am J Respir Crit Care Med.

[13] Alsma J., van Saase J., Nanayakkara P.W.B. (2017). The power of flash mob research: conducting a nationwide observational clinical study on capillary refill time in a single day. Chest.

[14] von Elm E., Altman D.G., Egger M. (2007). The Strengthening the Reporting of Observational Studies in Epidemiology (STROBE) statement: guidelines for reporting observational studies. Lancet.

[15] Dallal G.E. (1988). LOGISTIC: a logistic regression program for the IBM PC. Am Stat.

[16] DeLong E.R., DeLong D.M., Clarke-Pearson D.L. (1988). Comparing the areas under two or more correlated receiver operating characteristic curves: a nonparametric approach. Biometrics.

[17] Mandrekar J.N. (2010). Receiver operating characteristic curve in diagnostic test assessment. J Thorac Oncol.

[18] Ghasemi A., Zahediasl S. (2012). Normality tests for statistical analysis: a guide for non-statisticians. Int J Endocrinol Metab.

[19] Dall'Ora C., Griffiths P., Hope J., Briggs J., Jeremy J., Gerry S. (2021). How long do nursing staff take to measure and record patients' vital signs observations in hospital? A time-and-motion study. Int J Nurs Stud.

[20] Kuit M., Veldhuis L.I., Hollmann M., Nanayakkara P., Ridderikhof M. (2023). Recognition of critically ill patients by acute healthcare providers: a multicenter observational study. Crit Care Med.

[21] Brabrand M., Hosbond S., Folkestad L. (2011). Capillary refill time: a study of interobserver reliability among nurses and nurse assistants. Eur J Emerg Med.

[22] Bansal V., Schuchert V.D. (2006). Jaundice in the intensive care unit. Surg Clin North Am.

[23] Asim M., Alkadi M.M., Asim H., Ghaffar A. (2019). Dehydration and volume depletion: how to handle the misconceptions. World J Nephrol.

[24] Holland M., Kellett J. (2022). A systematic review of the discrimination and absolute mortality predicted by the National Early Warning Scores according to different cut-off values and prediction windows. Eur J Intern Med.

[25] Muralitharan S., Nelson W., Di S., McGillion M., Devereaux P.J., Barr N.G. (2021). Machine learning-based early warning systems for clinical deterioration: systematic scoping review. J Med Internet Res.

[26] Guarino M., Maroncelli G., Perna B., Baldin P., Ghirardi C., Zanotto A. (2025). Comparison of four non-invasive tools for predicting sepsis and septic shock mortality: a prospective cohort study. Clin Exp Emerg Med.

[27] Hernández G., Ospina-Tascón G.A., Damiani L.P., Estenssoro E., Dubin A., Hurtado J. (2019). Effect of a resuscitation strategy targeting peripheral perfusion status vs serum lactate levels on 28-day mortality among patients with septic shock: the ANDROMEDA-SHOCK randomized clinical trial. JAMA.

[28] Jones D., Bellomo R., DeVita M.A. (2009). Effectiveness of the medical emergency team: the importance of dose. Crit Care.

[29] Babyak M.A. (2004). What you see may not be what you get: a brief, nontechnical introduction to overfitting in regression-type models. Psychosom Med.

[30] Brunker C., Harris R. (2015). How accurate is the AVPU scale in detecting neurological impairment when used by general ward nurses? An evaluation study using simulation and a questionnaire. Intensive Crit Care Nurs.

